# Supplementary material for: The Anti-Obesity Effect of Fish Oil in Diet-Induced Obese Mice Occurs via Both Decreased Food Intake and the Induction of Heat Production Genes in Brown but Not White Adipose Tissue
Source: Int J Mol Sci. 2024 Dec 31;26(1):302. doi: 10.3390/ijms26010302 (PMC11719521; doi:10.3390/ijms26010302)
Supplement: Supplementary file 1 [file ijms-26-00302-s001.zip › Supplementary File S1.pdf]

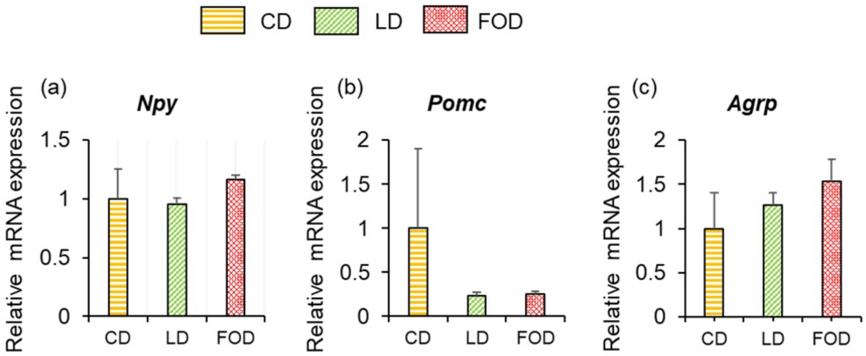

Supplementary Figure S1. No changes in gene expression related to feeding regulation were observed in the hypothalamus. (a) *Npy*; (b) *Pomc*; (c) *Agrp*. The data are presented as the mean ± standard error of the mean, n = 3 animals per group. CD, control diet; LD, lard-based high-fat diet; FOD, fish-oil based high-fat diet; *Npy*, neuropeptide Y; *Pomc*, Pro-opiomelanocortin; *Agrp*, Agouti-related peptide.

Supplementary Table S1. Typical Fatty Acid Profiles of experimental diets.

	**CD**	**LD**	**FOD**
g/kg			
Lard	0	240	0
Menhaden Oil	0	0	240
Soybean Oil	40	15	15
Fatty Acid Profiles (g/kg)			
C10, Capric	0	0.1	0
C12, Lauric	0	0.2	0
C14, Myristic	0	2.8	16.6
C15	0	0.2	1.1
C16, Palmitic	4.1	47.9	37.1
C16:1, Palmitoleic, ω-9	0	3.3	23.4
C16:2, ω-4	0	0	3.9
C16:3, ω-9	0	0	3.6
C16:4, ω-4	0	0	3.7
C17	0	0.9	0.9
C18, Stearic	1.5	26.0	6.8
C18:1, Oleic, ω-9	9.2	82.3	26.5
C18:2, Linoleic, ω-6	20.7	66.3	12.3
C18:3, Linolenic, ω-3	3.0	4.3	4.7
C18:4, Stearidonic	0	0	7.4
C20, Arachidic	0.1	0.4	0.5
C20:1	0.1	1.5	3.6
C20:2	0	1.9	0.5
C20:3, ω-6	0	0.3	1.0
C20:4, Arachidonic, ω-6	0	0.7	5.0
C20:5, Eicosapentaenoic, ω-3	0	0	34.1
C21:5, ω-3	0	0	1.8
C22, Behenic	0.1	0	0.3
C22:1, Erucic	0	0	0.7
C22:4, Clupanodonic, ω-6	0	0	0.5

C22:5, Docosapentaenoic, ω-3	0	0.2	6.7
C22:6, Docosahexaenoic, ω-3	0	0	24.6
C24, Lignoceric	0.1	0	1.5
C24:1	0	0	0.5
ω-6 (g)	20.7	67.3	18.8
ω-3 (g)	3.0	4.5	77.6
ω-6: ω-3 ratio	7.0	14.9	0.2

CD, control diet; LD, lard-based high-fat diet; FOD, fish oil-based high-fat diet.
